# Blood cancer care in a resource limited setting during the Covid-19 outbreak; a single center experience from Sri Lanka

**DOI:** 10.1371/journal.pone.0256941

**Published:** 2021-09-17

**Authors:** Saman Hewamana, Thurairajah Skandarajah, Chathuri Jayasinghe, Samadhi Deshapriya, Dhananjani Senarathna, Gehan Arseculeratne, Mahesh Harischandra, Gnani Somasundaram, Vadivelu Srinivasan, Surjit Somiah, Nihal Munasinghe, Sangeetha Hewawasam, Lalith Ekanayake, Rohini Wadanamby, Geethani Galagoda, Thet Thet Lin, Jayantha Balawardena

**Affiliations:** 1 Clinical Haematology Unit, Lanka Hospital, Colombo, Sri Lanka; 2 National Cancer Institute, Colombo, Sri Lanka; 3 University of Sri Jayewardenepura, Colombo, Sri Lanka; 4 Lanka Hospitals, Colombo, Sri Lanka; 5 Lanka Hospital Diagnostics, Colombo, Sri Lanka; 6 Faculty of Health and Life Sciences, Coventry University, Coventry, United Kingdom; 7 Sir John Kotelawala Defence University, Werahera, Colombo, Sri Lanka; Ohio State University Wexner Medical Center Department of Surgery, UNITED STATES

## Abstract

**Background:**

The Covid-19 pandemic has caused significant morbidity and mortality among patients with cancer. Most countries employed measures to prevent spread of Covid-19 infection which include shielding, quarantine, lockdown, travel restrictions, physical distancing and the use of personal protective equipment. This study was carried out to assess the change in patient attendance and the efficacy of newly implemented strategies to mitigate the impact of Covid-19 on services at the Lanka Hospital Blood Cancer Centre (LHBCC) in Colombo, Sri Lanka.

**Methodology:**

Telephone consultation, infection control, personal protective measures and emergency admission policy were implemented with the aim of having a Covid-19 free ward and to prevent cross-infections. This descriptive cross-sectional study was conducted with 1399 patient episodes (in-patient care or day-case review). We analysed patients treated as in-patient as well as day-case basis between 01^st^ April 2020 and 31^st^ December 2020.

**Results:**

There were 977 day-case based episodes and 422 in-patient based episodes. There was a 14% drop in episode numbers compared to same period in 2019. There was no cross infection and no patients with Covid-19 related symptoms or positive test results entered the LHBCC during the study period.

**Conclusion:**

Services in blood cancer care were maintained to prevent late stage presentation and adverse outcome. Measures implemented to prevent Covid-19 were effective to allow continuation of treatment. This study highlights the importance of implementing strict protocols, clinical screening, use of appropriate personal protective equipment in delivering blood cancer care during the Covid-19 pandemic. This is the only documented study relating to outcome and successful applicability of measures to prevent spread of Covid-19 infection and maintaining services among blood cancer patients in Sri Lanka.

## Introduction

Haemato-Oncology patients have a higher risk of acquiring infections. It has been known for decades that infection is the main cause of death in patients with blood cancer [1]. Due to complexity of haematological malignancies, complications due to treatment, comorbidities associated with increasing age, these patients require multidisciplinary strategies and care from staff experienced in blood cancer management.

Coronavirus disease 19 (Covid-19) is caused by Severe Acute Respiratory Syndrome Corona Virus-2 (SARS-CoV-2) infection [2]. Three-phase approach in the bed site is proposed in handling patients with Covid-19 infection [3]. It has dramatically changed global health care deliveries, particularly cancer care. Covid-19 has an adverse effect on patient care in terms of delay in diagnosis as well as treatment in addition to mortality associated with SARS-CoV-2 infection. Poor out come with persistence of virus in patients with haematological malignancies is reported [4, 5]. Furthermore Covid-19 is shown to cause various haematological abnormalities [6]. This has brought new challenges to haematologists during the Covid-19 pandemic [7] more so in the resource limited setting. Low-income countries are less prepared to face the pandemic with fragile heath care systems hence rapid control measures are needed to prevent catastrophic consequences [8].

SARS-CoV-2 is spread via direct or indirect contact via respiratory droplets, saliva, aerosols or fomites [9] while crowded indoor environments are a particularly high-risk for transmission of infections [10]. World Health Organization (WHO) has made recommendations for developing and implementing control measures to prevent transmission [11].

Sri Lanka is a developing country with diverse healthcare structure. Lanka Hospital Blood Cancer Centre (LHBCC) is a dedicated unit established for the purpose of treating blood related disorders in Colombo, Sri Lanka. Several new measures were implemented in April 2020 to reduce Covid-19 infection among patients and staff. The aim of the study was to analyze, the effect of Covid-19 pandemic on the patient numbers in LHBCC and the efficacy of the implemented measures with regards to the outcome of Covid-19 prevention among the patients and staff at the LHBCC.

## Methodology

### Ethical considerations

Study was considered as a quality improvement activity and approval was obtained from the Lanka Hospitals medical research and the ethics committee for the collection and analysis of anonymized data. Informed consent was obtained from all patients.

### Site, subjects and implemented measures

This study was conducted at LHBCC. This is the first dedicated blood cancer centre in Sri Lanka which consists of designated in-patient (8-bedded ward), day-case (4-bedded space) facility, staff and a strategy to treat blood cancers using treatment protocols from high-income countries.

In-patients were not in contact directly or indirectly with patients who attending the day-case facility. There were separate sets of nurses for in- and day- patients for each shift. In addition, LHBCC was used for training purposes of first Haemato-Oncology trainees from government subsidised hospitals.

All patients treated as in-patient and day-case basis between 01^st^ April 2020 to 31^st^ December 2020 at the LHBCC were used in the study. We also collected similar data from all patients treated as in-patient and day-case basis between 01^st^ April 2019 to 31^st^ December 2019 at LHBCC for comparative purposes.

Following measures were implemented from 01.04.2020 to 31.12.2020.

1. Strict shielding / isolation advice was given to all patients with a diagnosis of blood cancer on systemic chemotherapy or on supportive care.
2. All patients were actively monitored via telephone after discharge to lower patient attendance without compromising care.
3. Colony stimulating factors (CSF) and oral antibacterial prophylaxis were used to minimize patients coming in to the hospital with neutropenic sepsis.
4. A triage system was implemented to minimize cross-infections. All planned admissions and day-case patients were reviewed via a telephone consultation on the day before and any patient with fever, respiratory symptoms, recent travel to outbreak areas or those with a contact history with positive patients were offered screening or delay non-essential appointments.
5. A strict no visitor policy was implemented for day-cases while single by-stander policy was in place for in-patients.
6. All emergency admissions were admitted to a dedicated separate section in the main hospital with an isolation facility.
7. Infection control training was provided to all clinical staff in addition to surgical / KN95 masks and eye protection measures.
8. All staff members were advised to measure their own body temperatures before work and promptly report any symptoms of upper respiratory tract infection; any one with symptoms suspicious of Covid-19 infection was screened with PCR testing.
9. Frequent disinfection of equipment and installation of protective shields were implemented to maximise environmental control and to reduce droplet transmission.

### Statistical analysis

Descriptive cross-sectional study was conducted with 1399 (422 in-patient and 977 day-case) episode participants, using a non-probability convenience sampling method. We analysed patients treated as in-patient and day-case basis between 01^st^ April 2020 and 31^st^ December 2020.

To obtain a thorough understanding of the sample of patients considered in the study, a comprehensive descriptive analysis was conducted initially. Clinical and demographic characteristics such as the type of the disease, neutrophil count, number of neutropenic episodes, number of non-neutropenic episodes, in-patient episodes, day-case episodes, number reviewed in-person and over the phone and according to disease type, age of the patient at presentation and gender were considered. Both numerical and graphical analyses were conducted to conduct an in-depth analysis using R software. To statistically compare proportions of various episodes like in-patient and day-case episodes that have occurred between 01^st^ April 2020 and 31^st^ December 2020 with the same period in the previous year, statistical hypothesis tests for comparing two proportions were conducted. Further to this, proportions of occurrences of telephone and in-person reviews were also compared between the two periods. It was also of interest to test various hypotheses related to percentage increase/decrease observed in different types of episodes, total number of episodes, different types of reviews conducted and total reviews conducted. To test these hypotheses statistical tests for testing one binomial proportion were utilized.

The distribution of age of the whole sample and also when considered, the two groups separately, is negatively skewed. Hence, median is used for analysis (S3 File).

For a two-tailed test of two proportions following null and alternative hypotheses were considered: H_0_: p_1_ = p_2_ versus H_1_: p_1_ ≠ p_2,_ where p1 and p2 are the proportions of events in populations 1 and 2, respectively. In the case of a one-tailed test of two proportions following null and alternative hypotheses were considered: H_0_: p_1_ = p_2_ versus H_1_: p_1_ > p_2,_ where p1 and p2 are as defined above. Similarly, for a two-tailed test of a single proportion the following null and alternative hypotheses were taken into consideration: H_0_: p = p_0_ versus H_1_: p ≠ p_0_ where p is the population proportion and p_0_ is the hypothesized value. For all tests 0.05 was taken as the level of significance. All statistical hypothesis tests were conducted using R software.

## Results

There were 1399 visits to the LHBCC. Forty seven percent were 60 years or over. Seventy percent were reviewed on the day-case basis and forty three percent were females. Plasma cell disorders (PCD), Lymphoproliferative disorders (LPD), Acute Myeloid Leukaemia (AML), Acute Lymphoblastic Leukaemia (ALL), Myelodysplastic Syndrome (MDS) and other disorders accounted for 33%, 27%, 11%, 3%, 11%, and 15% respectively.

The number of episodes reviewed in the LHBCC during the study period according to age, gender disease type were as follows. A) Age ≥ 60, In-patient (n = 185), Day-case (n = 553) and < 60, In-patient (n = 237) and Day-case (n = 424); B) Gender male, In-patient (n = 242), Day-case (n = 557) and female, In-patient (n = 180) and Day-case (n = 420); C) Disease type, PCD, In-patient (n = 57), Day-case (n = 405); LPD, In-patient (n = 113), Day-case (n = 269); AML, In-patient (n = 46), Day-case (n = 106); ALL, In-patient (n = 12), Day-case (n = 35); MDS, In-patient (n = 70), Day-case (n = 81); Other, In-patient (n = 124), Day-case (n = 81).

There were 324 neutropenic episodes during the study period. Thirty percent of those episodes were treated as in-patient due to neutropenic sepsis.

**Fig 1:**
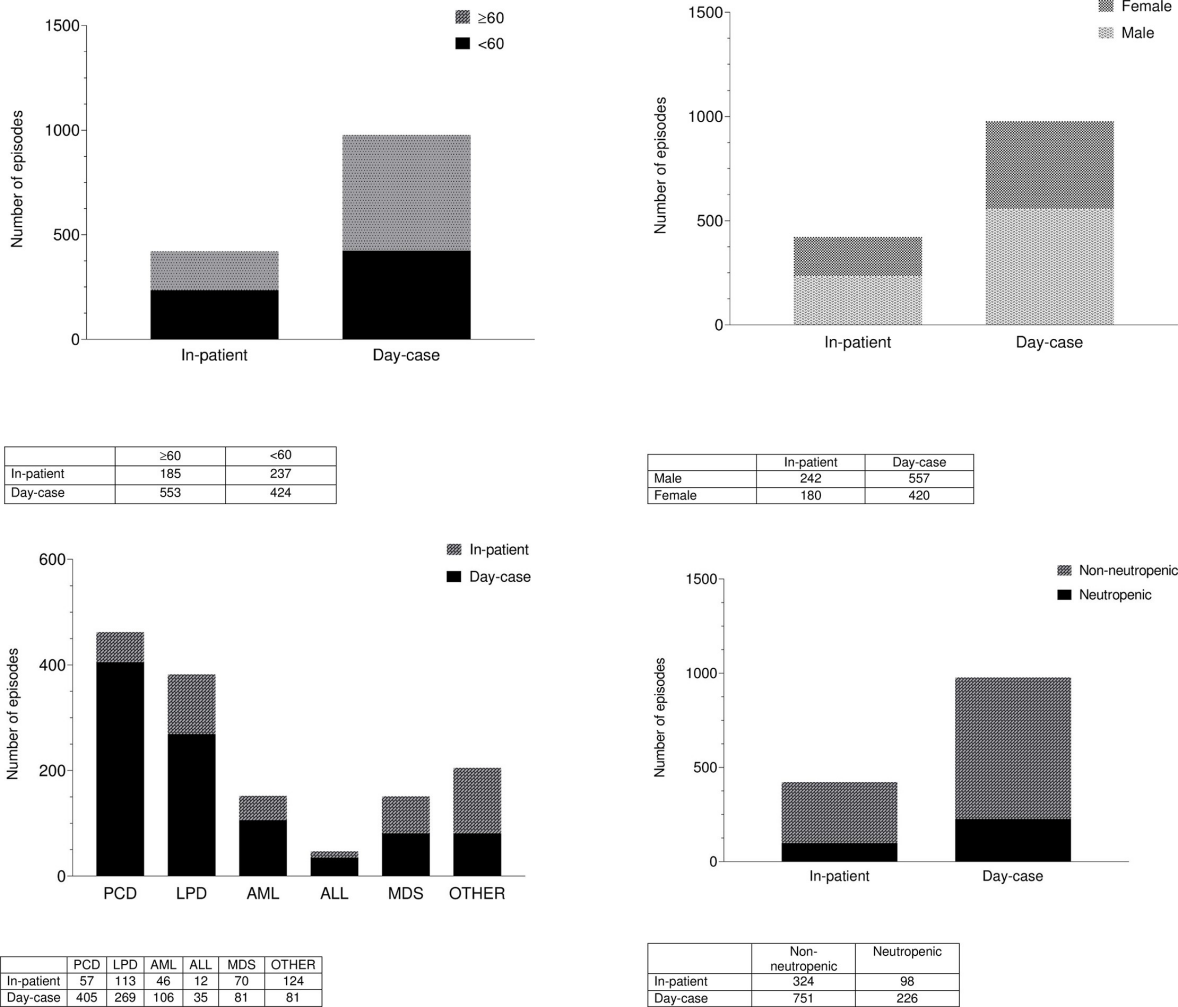
Patients reviewed as in-patient and as day-case according to A) age, B) gender, C) disease type and D) neutrophil count.

Clinical and demographic characteristics of patients are summarized in Table 1 and Fig 1.

**Table 1:** Clinical and demographic characteristics of total episodes (n = 1399) reviewed during the study period.

	In-patient care	Day-case	p-value
**Total episodes**	422	977	0.000
**Gender (male/female)**	242 / 180	557 / 420	0.681
**Median Age (IQR)**	59 (31.25)	61 (23.5)	0.0001
**Disease Type**			
Plasma Cell Disorders	57	405	0.000
LPD	113	269	0.794
AML	46	106	1.000
ALL	12	35	0.523
MDS	70	81	0.000
Other	124	81	0.000
Neutropenic episodes	98	226	1.000
Covid-19 PCR performed	26		
Telephone Consultations		353	

**Fig 2:**
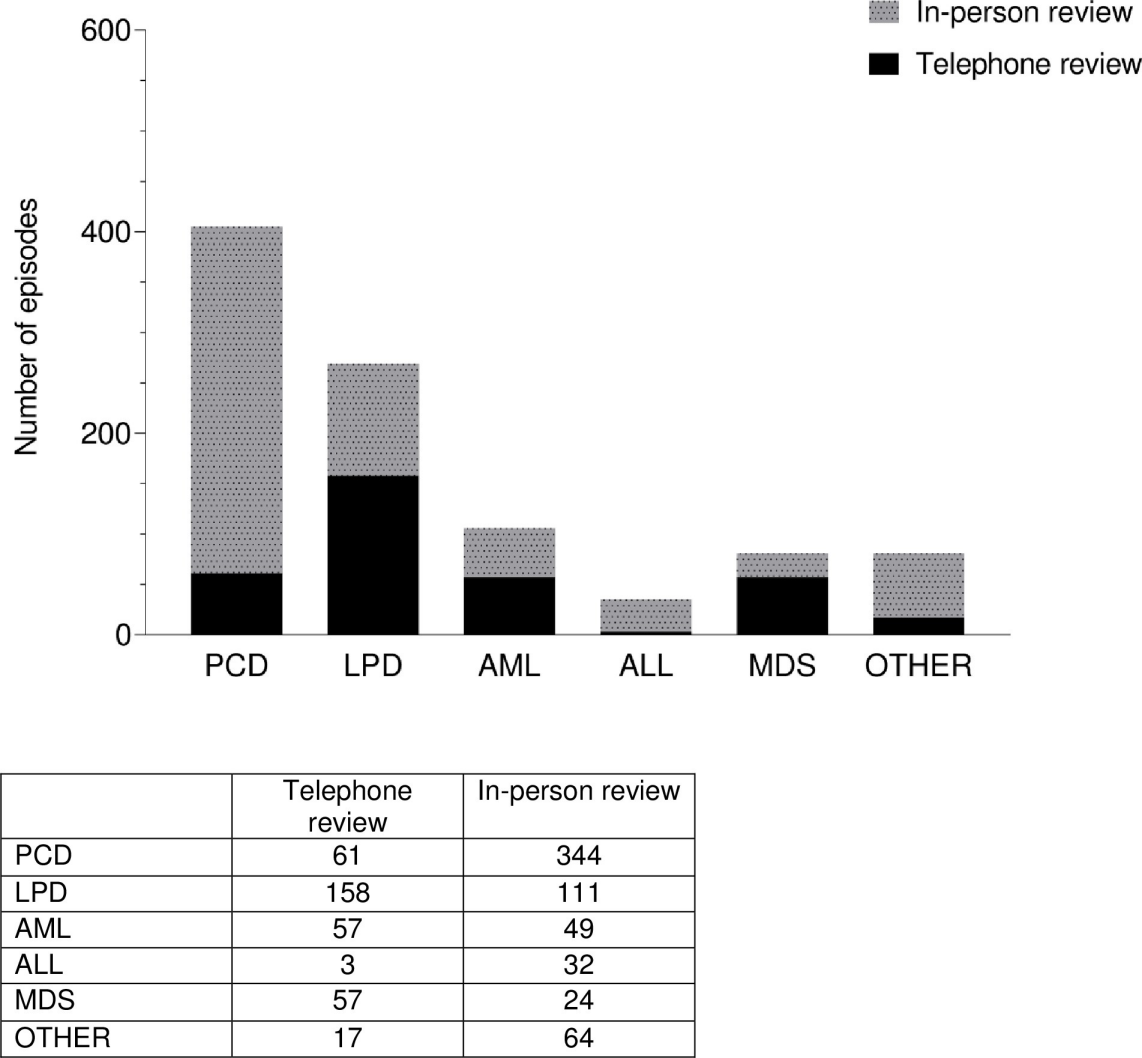
Patients reviewed on day-case basis in person (n = 624) and telephone review (n = 353) during the study period according to disease type.

Thirty six percent (353) of day-case based reviews were done over the phone during the study period. The percentage of PCD, LPD, AML, ALL, MDS and other disorders reviewed were 15%, 59%, 53%, 8%, 70% and 20% respectively (Fig 2).

During the same period in 2019 we have treated a total of 1633; 574 as in-patient and 572 in-person in the day-case and 487 over telephone consultations. During the study period compared to the same period in 2019, 14% decrement in total was observed with a 95% confidence interval (CI) of (12.66%, 16.12%). Among these a statistically significant decrement (26% with 95% CI (22.91%, 30.30%) and p-value 0.002) was observed during the study period compared to same period in 2019 with regards to in-patient episodes whereas he increment observed in 2020 compared to 2019 in day-case (8% with a 95% CI of (6.21%, 9.52%) and p-value 0.002) was also found to be statistically significant under 0.05 significance level (Tables 2 and 3).

**Table 2:** In-patient, day- case based and total episodes in 2020 compared to 2019.

	2019 count	Proportion	2020 count	Proportion	% decrement/increment (95% CI)
In-patient	574	0.35	422	0.30	26% (22.91%, 30.30%)
Day-case	1059	0.65	977	0.70	8% (6.21%, 9.52%)
Total Episodes	1633		1399		14% (12.66%, 16.12%)

**Table 3:** Proportional comparison of in-patient, day- case based and total episodes in 2020 compared to 2019.

	Proportion comparison between 2019 and 2020 p-value	Conclusion	% decrement/ Increment Significance p-value	Conclusion
In-patient	0.003	significant difference	0.441	%decrement is not significantly different from 25%
	0.002	Significantly lower in 2020		
Day-case	0.004	significant difference	0.778	%increment is not significantly different from 8%
	0.002	Significantly higher in 2020		
Total Episodes			0.721	%decrement is not significantly different from 14%

**Fig 3:**
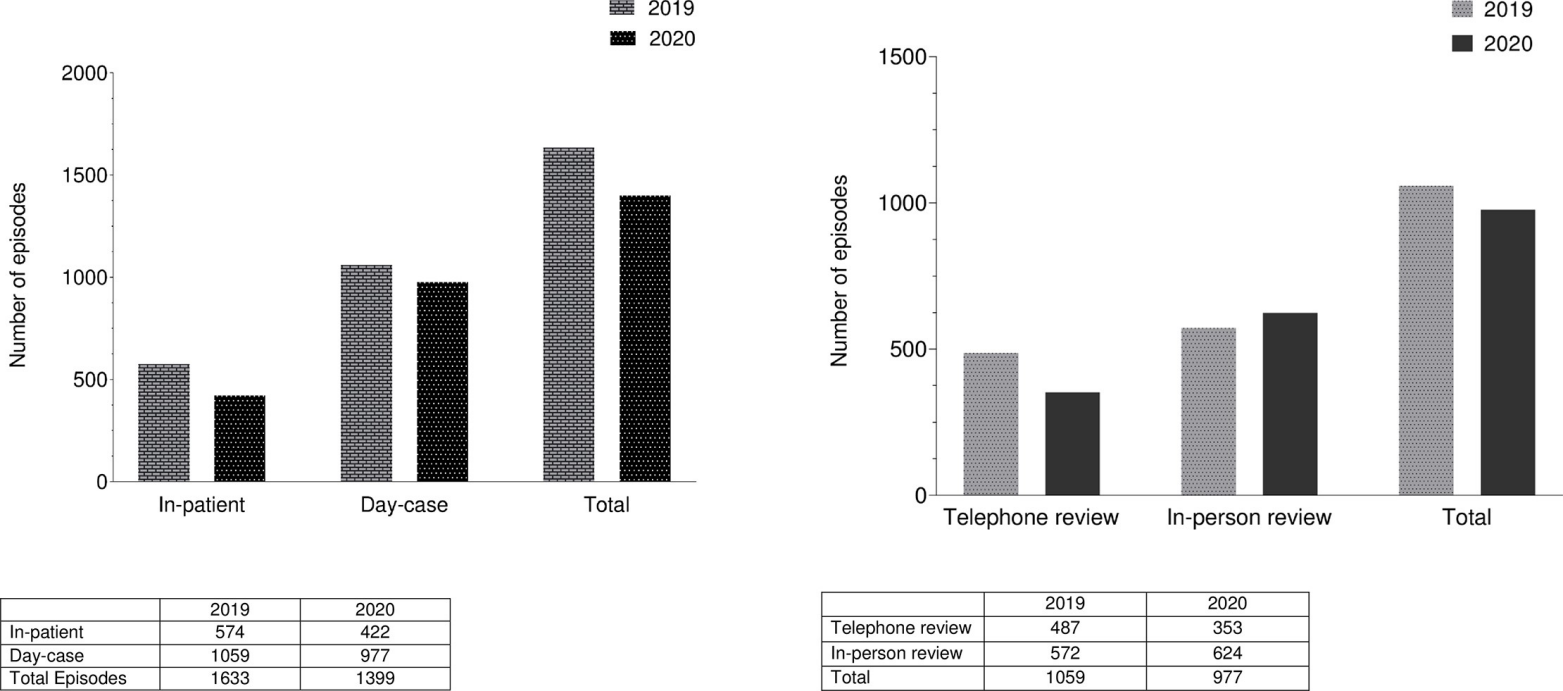
A). Patient episodes reviewed on in-patient and day-case basis in 2020 compared to in-patient and day-case basis in 2019. B) Day-case episodes reviewed in-person and on the telephone in 2020 compared to in-person and telephone review in 2019.

Thirty six percent of day-case based episodes were reviewed over the phone in the study period compared to 46% in the same period in 2019 (Fig 3). A decrement of day-case based reviews in the study period compared to the same period in 2019 in total was observed as 8% with a 95% CI of (6.21%, 9.52%). There was a statistically significant drop of 28% (with a 95% CI of (23.59%, 31.71%) and p-value 0.000) in the telephone consultations but in-person reviews were up by 9% (with a 95% CI of (6.86%, 11.75%) and this increment observed was found to be statistically significant (p-value 0.000) (Tables 4 and 5).

**Table 4:** Day-cases based episodes reviewed over the phone during the study period compared to 2019.

	2019 count	Proportion out of total	2020 count	Proportion out of total	% decrement/Increment (95% CI)
Telephone review	487	0.46	353	0.36	28% (23.59%, 31.71%)
In person review	572	0.54	624	0.64	9% (6.86%, 11.75%)
Total	1059		977		8% (6.21%, 9.52%)

**Table 5:** Proportional comparison of day-cases based episodes reviewed over the phone during the study period compared to 2019.

	Proportion comparison between 2019 and 2020 p-value	Conclusion	% decrement/ Increment Significance p-value	Conclusion
Telephone review	0.000	significant difference	0.209	%decrement is not significantly different from 25%
	0.000	Significantly lower in 2020		
In person review	0.000	significant difference	0.487	%increment is not significantly different from 10%
	0.000	Significantly higher in 2020		
Total			0.778	%decrement is not significantly different from 8%

24 patient tests performed as directed by LHBCC. There were no infections recorded in patients who got admitted or were cared in the day-care setting or among the staff in the LHBCC during the study period. A total of 5 staff with suspected Covid-19 symptoms or contact history were isolated. However, none of them had a positive PCR result. Two patients identified with suspected Covid-19 symptoms, tested positive on the out-patient basis admitted to a separate ward with isolation facilities. Both were treated at quarantine centers outside the main hospital before returning for follow up in LHBCC.

In addition, one out-sourced janitorial staff member had symptomatic Covid-19 diagnosed during work and was treated at an isolation facility.

## Discussion

Patients on systemic chemotherapy are at a higher risk of death with Covid-19 infection. Higher mortality is reported in Haemato-Oncology patients compared to those with solid cancers [5, 12] which is higher than 1.4%, originally reported from China [2]. In addition, Covid-19 caused devastating effect on cancer patients due to delayed diagnosis and treatment [13]. Implications of continuing treatment has to be weighed against the implications on outcome due to treatment delay. There are published guideline about blood cancer care during Covid-19 pandemic [14, 15]. Delay in diagnosis and treatment is likely to cause a spike in late stage cancer presentation in the future.

Sri Lanka is a developing country with a diverse health care system. Unlike high-income countries, hospitals in Sri Lanka are likely to have different approaches in managing the same disease and also significant heterogeneity exists with regard to diagnostic and treatment facilities, access to trained personnel and supportive care. LHBCC was established in collaboration with colleagues from government subsidised hospitals. We have previously published data on the successful application of British treatment protocols in acute myeloid leukaemia [16], in Hodgkin Lymphoma [17] and other haematological malignancies (manuscript in preparation). Covid-19 incidence is on the rise in Sri Lanka [18]. LHBCC also suffered the impact of Covid-19 outbreak and had a challenge to balance the risk and benefit of continuing treatment and keeping the centre free of this infection.

We had to make the difficult decision of treating aggressive diseases while at the same time minimizing the risk of exposure to Covid-19 and avoiding medication related toxicity with limited resources. We redesigned the LHBCC and introduced stringent precautions against viral transmission which also included hygienic behavior outside the work place for staff and patients.

Our study showed 14% drop in episodes after Covid-19 epidemic compared to pre-Covid -19 era. Delay in treatment of patients with aggressive haematological malignancies can cause detrimental effects on the outcome [19]. In addition, as published previously it is likely that Covid-19 pandemic will have an impact on the morbidity and mortality in cancer patients in the future [13]. Willan et al, reported 53% less diagnosis due to reduction in patient numbers and doctors performing less procedures [7]. Maintaining 86% episodes in LHBCC was considered as an important step in preventing inferior out come and late relapses due to sub optimal treatment in the future. There was a higher drop in in-patient episodes but increase in the proportion of episodes in the day-case. This may be related to drop in total patient numbers, less admissions due to better day-case, telephone advice or better preventive measures against complications related to treatment. A larger study may be needed to investigate it further. Although we cannot generalize our conclusions about the cancer care to the rest of the country.

Covid-19 can spread from contaminated surfaces, respiratory droplets or other body fluids via respiratory or non-respiratory surfaces [20, 21]. Requesting patients to be at their homes to avoid viral transmission was highly effective. Behavior modification can reduce the transmission, prevent disease spread and contribute to release the overwhelmed health care system [22]. Telephone screening has been shown to be effective in cancer care as reported previously [23–25]. We have had an active telephone consultation facility since 2013. Thirty six percent of LHBCC day-case consultations were done via telephone during the study period. However, forty five percent of consultations were carried out using the telephone facility during the same period in 2019. This data shows that we had an established effective telephone consultation strategy before Covid-19 pandemic to prevent infections among blood cancer patients. Keeping high risk patients at home was a very effective and may be the most effective strategy in keeping patients safe. Furthermore, we utilized measures with proven benefit such as primary prophylaxis with CSF [26] and oral antibacterial medications to minimize patients coming in to the hospital with neutropenic sepsis. There were 324 episodes with neutropenia and 30% of those ended up with neutropenic sepsis but with no mortality.

We implemented an additional telephone consultation service for risk assessment with every scheduled admission for in-patient care or for day-case treatment to keep LHBCC a Covid-19-free area. Telephone triage before attending to medical clinic has been shown to be effective in managing patients during the Covid-19 pandemic [27]. Two positive PCR reports were noted on patients; one patient identified with suspected Covid-19 symptoms, tested on the outpatient basis and another with similar symptoms admitted to a different ward with isolation facilities. No patient with Covid-19 related symptoms or positive results entered LHBCC making this a highly effective strategy.

Health professionals are more vulnerable to Covid-19 and also can pass it on to other patients due to the nature of their profession [28]. It is of paramount importance to protect them from clinical or sub-clinical infections [29]. Using face shields/goggles, regular decontamination of the patient’s surroundings, use of N95 and use of single use surgical gowns have been shown to protect separate staff from contacting Covid-19 [30]. One of our out-sourced janitorial staff members had symptomatic Covid-19 but neither staff nor patients contacted virus from this member. We believe our data reflects the efficacy of stringent measures we employed to prevent cross infections in the ward. The threat of infection among patients and staff had to be taken seriously if we were to continue safe practice in the LHBCC. Several studies have shown the difference in morbidity and mortality according to availability of nursing and medical staff [31, 32]. Our center has a resident full-time consultant hematologist and during the time of the epidemic we restructured the LHBCC to maintain a higher patient nurse ratio. We believe this to be one of the key elements in delivery of a safe, secure and less stressful practice.

Routine screening and PCR / RAT testing have been shown to reduce infection rate [33, 34]. However, we did not routinely check patients for SARS-CoV-2 by PCR and this was reserved for high risk patients mainly due to financial and practical reasons. However, we realise this may be a requirement if the future if infection rate continues to rise or if there is an outbreak in LHBCC. We have included RAT testing in the proposed advanced admission and outpatient care policy in LHBCC (**S4 File**).

We had a very successful control of Covid-19 infection at LHBCC and we believe the success was due to stringent precautionary measures adapted at the LHBCC. Others have reported similar data from the developed world following strict preventative measures [19]. The measures implemented to prevent Covid-19 were effective to allow continuing treatment. However, it is not possible to differentiate or assess the efficacy of implemented individual measures.

We did not delay in commencing intended treatment due to Covid-19 pandemic and this study demonstrates that blood cancer patients can be managed safely even in a resource limited setting using high clinical awareness, education, telephone screening and performing PCR on symptomatic patients. Urgent chemotherapy should not be delayed and our data suggests patients and staff can be kept Covid-19 free despite continuing on aggressive treatment and having limited resources. The success we encountered may reflect staff and patient awareness and commitment. Clear guidelines, improved communication and strong leadership are necessary to protect patients and staff from Covid-19 infection.

### Limitations

We recognise limitations in this study. This study was a single-centre study and further studies are necessary with a large multi-centre samples before reaching definitive conclusions. There are certain disadvantages particularly related to financial implications related to increase cost of treatment and drop in income due to smaller number of patients and psychological impact of Covid-19 and implemented measures on healthcare workers and patients. In addition, the results might not be generalized due to differences socioeconomic backgrounds in other centres. Larger studies may be necessary to address above limitations.

## Conclusion

Services in blood cancer care were maintained to prevent late stage presentation and adverse outcome. However there was a significant drop in number of patients attending blood cancer care during the Covid-19 pandemic. Measures implemented to prevent Covid-19 were effective to allow continuation of treatment. This study highlights the importance of implementing strict protocols, clinical screening, use of appropriate personal protective equipment in delivering blood cancer care during the Covid-19 pandemic. This is the only documented study relating to outcome and successful applicability of measures to prevent spread of Covid-19 infection and maintaining services among blood cancer patients in Sri Lankan.

## Supporting information

S1 File Covid-19 screening check list 1. (DOCX)

S2 File Covid-19 screening check list 2. (DOCX)

S3 File Age distribution. (DOCX)

S4 File Proposed advanced admission and out-patient care policy in LHBCC during Covid-19 pandemic. (DOCX)

S5 File Admission. (XLSX)

S6 File Day-case. (XLSX)
