# Supplementary material for: Blood cancer care in a resource limited setting during the Covid-19 outbreak; a single center experience from Sri Lanka
Source: PLoS One. 2021 Sep 17;16(9):e0256941. doi: 10.1371/journal.pone.0256941 (PMC8448336; doi:10.1371/journal.pone.0256941)
Supplement: S1 File — (DOCX) [file pone.0256941.s001.docx]

**COVID-19** screening check list 1

| 01 | Current address: from an endemic area es) (**If yes**) specify……………………………………………. |  | Yes No   \|  \| \| --- \|  \|  \| \| --- \| |
| --- | --- | --- | --- | --- | --- |
| 02 | Travel history from abroad within 1 month  (**If yes**)specify……………………………………………… |  | Yes No   \|  \| \| --- \|  \|  \| \| --- \| |
| 03 | Travel to an endemic/isolated area, within 1 month  (**If yes**)specify ….………………………………………… |  | Yes No   \|  \| \| --- \|  \|  \| \| --- \| |
| 04 | Contact history with a Covid-19 positive patient or quarantined person  **If yes,** |  | Yes No   \|  \| \| --- \|  \|  \| \| --- \| |
| 4.1 | Availability of certification of successful completion of COVID-19 self-quarantine process |  | Yes No   \|  \| \| --- \|  \|  \| \| --- \| |
| 4.2 | Time period of quarantine |  | from…../…../2020 to…../…../2020 |
| 4.3 | Place of quarantine |  | home/hotel/work place/ quarantine centre |
| 4.4 | Reason for quarantine |  |  |
|  | Traveling from a foreign country (.....)    Close contact with Positive Covid-19 person (….)    Close contact with suspected person (….)  Suspected Covid-19 person (.…)  Clinically suspected patient (.…) |  |  |
|  |  |  |  |
| 5. | History of family members related to above 1,2,3,4  **(If yes**) specify …………………………………………… |  | Yes No   \|  \| \| --- \|  \|  \| \| --- \| |

***Assessing COVID risk***

When all questions are negative (0) – Low risk

All others (>0) – High risk
