# Supplementary material for: Blood cancer care in a resource limited setting during the Covid-19 outbreak; a single center experience from Sri Lanka
Source: PLoS One. 2021 Sep 17;16(9):e0256941. doi: 10.1371/journal.pone.0256941 (PMC8448336; doi:10.1371/journal.pone.0256941)
Supplement: S2 File — (DOCX) [file pone.0256941.s002.docx]

**COVID-19** screening check list 2

| 01 | Last date of chemotherapy | : |
| --- | --- | --- |
| 02 | Last FBC report | : |
|  |  |  |
|  |  |  |
| 03 | Current symptoms  Fever | Yes No   \|  \| \| --- \|  \|  \| \| --- \| |
|  | Sore throat    Cough | Yes No   \|  \| \| --- \|  \|  \| \| --- \|     Yes No   \|  \| \| --- \|  \|  \| \| --- \| |
|  | Difficulty in breathing | Yes No   \|  \| \| --- \|  \|  \| \| --- \| |
|  | Diarrhea | Yes No   \|  \| \| --- \|  \|  \| \| --- \| |
| 04 | Previous history of chest infection | Yes No   \|  \| \| --- \|  \|  \| \| --- \| |
|  | **If yes,**  Duration  Last date of treatment | :  : |
