# Supplementary material for: Blood cancer care in a resource limited setting during the Covid-19 outbreak; a single center experience from Sri Lanka
Source: PLoS One. 2021 Sep 17;16(9):e0256941. doi: 10.1371/journal.pone.0256941 (PMC8448336; doi:10.1371/journal.pone.0256941)
Supplement: S3 File — (DOCX) [file pone.0256941.s003.docx]

| Age | N | Mean (StDev) | Median (IQR) |
| --- | --- | --- | --- |
| Admissions | 422 | 53.88 (19.65) | 59.00 (31.25) |
| HDU | 977` | 58.42 (16.87 | 61.00 (23.50) |


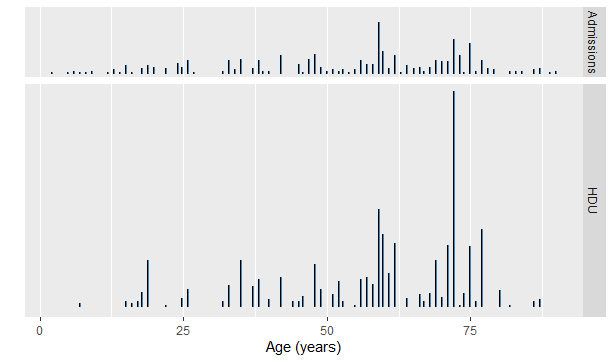


| Age | N | Mean (StDev) | Median (IQR) |
| --- | --- | --- | --- |
| All patients | 1399 | 57.05 (17.87) | 60.00 (25.00) |


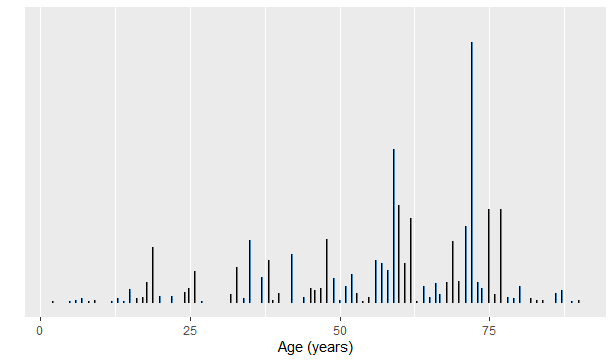


The distribution of age of the whole sample and also when considered, the two groups separately, is negatively skewed. Median was used to discuss the central tendency; interquartile range was used to discuss how dispersed (varied) the random variable is.
