# Supplementary material for: Blood cancer care in a resource limited setting during the Covid-19 outbreak; a single center experience from Sri Lanka
Source: PLoS One. 2021 Sep 17;16(9):e0256941. doi: 10.1371/journal.pone.0256941 (PMC8448336; doi:10.1371/journal.pone.0256941)
Supplement: S4 File — (DOCX) [file pone.0256941.s004.docx]

**Proposed advanced admission and out-patient care policy in LHBCC during COVID 19 pandemic**

**All asymptomatic patients**

1. 6009 is designated room for COVID 19 rapid antigen test (RAT) for Haematology/ Haemato-Oncology patients
2. Allow only one patient can stay in 6009 room at a time and no visitors
3. If RAT **negative** for COVID 19, proceed with Haemato-Oncology unit admission and outpatient care policy
4. If RAT **positive**, follow hospital policy

**All symptomatic patients**

1. Direct to Lanka Hospital ETU and do RAT
2. If RAT **negative**, admit to 5^th^ floor for PCR and planned care
3. Transfer to Haemato-Oncology unit with **negative** report
4. If RAT or PCR **positive**, follow hospital policy
